# Determinants of an unfavorable treatment outcome among tuberculosis patients in the Jimma Zone, Southwest Ethiopia

**DOI:** 10.1038/s41598-024-78084-0

**Published:** 2024-11-26

**Authors:** Berhane Megerssa Ereso, Mette Sagbakken, Christoph Gradmann, Solomon Abebe Yimer

**Affiliations:** 1 Department of Community Medicine and Global Health, Faculty of Medicine, Institute of Health and Society, University of Oslo, Oslo, Norway; 2 Department of Health Policy and Management, Faculty of Public Health, Institute of Health, Jimma University, Jimma, Ethiopia; 3 Department of Nursing and Health Promotion, Faculty of Health Sciences, OsloMet - Oslo Metropolitan University, Oslo, Norway; 4 Department of Microbiology, Institute of Clinical Medicine, University of Oslo, Oslo, Norway; 5 Coalition for Epidemic Preparedness Innovations (CEPI), Oslo, Norway

**Keywords:** Tuberculosis, Treatment outcomes, Determinants, Unfavorable outcome, Diseases, Health care, Medical research

## Abstract

Tuberculosis (TB) is a major public health challenge in Ethiopia. TB treatment outcomes were suboptimal compared to the expected target of the national TB control Program. The provision of standard anti-TB treatment is the primary component of the directly observed treatment, short-course strategy. The aim of this study was to assess the TB treatment outcomes and the determinants of an unfavorable treatment outcome. The study used a cross-sectional study design at baseline and record review to identify treatment outcomes. A total of 1,161 TB patients were recruited from eight randomly selected districts and one town administration in the Jimma Zone, Ethiopia. Treatment outcomes were grouped into favorable and unfavorable. Of the total participants, 86.9% had a favorable treatment outcome, and 5.7% an unfavorable treatment outcome. The rest were transferred out and not recorded cases. Women were more likely to experience an unfavorable treatment outcome [adjusted odds ratio (AOR) = 1.96, 95% CI 1.06, 3.64]. Patients who were perceived to not be stigmatized were less likely to have an unfavorable treatment outcome (AOR = 0.32, 95% CI 0.15, 0.73). Patients who had a monthly income of > 3,500 Ethiopian *birr* were less likely to have an unfavorable outcome than patients who did not have a regular income (AOR = 0.04, 95% CI 0.01, 0.45). The observed treatment success rate is lower than the World Health Organization’s target of successfully treating > 90% of detected TB cases. It is imperative to ensure that information, education and communication/behavior change communication strategies consider the needs of women and patients with perceived TB stigma. Furthermore, designing locally acceptable and affordable interventions may help to address the financial challenges of TB treatment adherence.

**Supplementary Information:**

The online version contains supplementary material available at 10.1038/s41598-024-78084-0.

## Introduction

Tuberculosis (TB) is a major public health challenge in the developing world. According to the global TB report of 2021, there were an estimated 10.6 million TB cases and 1.3 million deaths due to TB in 2022^1^. A majority (86%) of the estimated incidence of TB cases are found in the 30 highest TB burden countries, with^2^ Ethiopia being one of the high TB burden countries^3^. There was an estimated 140 per 100,000 population TB incidence rate in 2020, and 19 per 100,000 population TB mortality rate in 2019 in Ethiopia^4^.

The World Health Organization (WHO) has designed the End TB strategy to end the TB epidemic by 2035. The aim of the strategy is to reduce the global TB incidence and mortality rates by 90% and 95%, respectively, when compared to 2015^5^. One of the pillars of implementing the End TB strategy is treating all people with TB, including drug-resistant TB, and to enhance patient support. For the optimal treatment success rate, the WHO recommends the need to address patients and health system-related challenges affecting a successful treatment outcome^6,7^. Among others, locally acceptable and affordable interventions need to be implemented to identify and address the physical, economic, and sociocultural challenges to accessing TB treatment services. Special attention is required to address the needs of the poorest and most vulnerable groups. In addition, addressing gender-related issues, improving staff attitudes and enhancing communication between the patient and the provider are equally important^6,7^.

The factors associated with unfavorable TB treatment outcome have previously been studied in different parts of the world. These factors include old age, being female, urban residence, education levels, underlying diabetes mellitus, cigarette smoking, extra-pulmonary TB, a history of previous TB treatment, HIV infection, TB relapse, being sputum-smear positive, being unemployed, and being a newly diagnosed TB case^8–12^. A study from 2019 conducted at the Jimma University Medical Center (one of the study sites from the present study area) indicated that 11.7% of patients had an unfavorable treatment outcome^13^, while 88.3% had a favorable treatment outcome. This finding was lower than the End TB strategy target of achieving ≥ 90% treatment success rate^5^.

Although several studies were conducted in Ethiopia and elsewhere to assess TB treatment outcomes and associated factors, there was only one study from the Jimma Zone, Ethiopia that had assessed treatment outcome and associated factors of unfavorable treatment outcomes at the time of the present study^13^. However, as this study only focused on one urban University Medical Centre located in the Zonal capital, the findings of the study were less likely to be representative of the entire Jimma Zone that comprises semi-urban and rural districts. Treatment outcomes and associated factors may vary according to the local setting, including the socio-demographic and economic conditions as well as the literacy status of the population in the study area. TB treatment outcomes monitoring and analyzing the factors affecting these outcomes are important for effective TB control program evaluation, and for providing feedback at the various levels of the health system (woreda/district, zonal, regional, or national level). The aim of this study was to assess TB treatment outcomes and the associated factors of unfavorable treatment outcomes among TB patients in the Jimma Zone, Ethiopia.

## Methods

### Study setting and design

The study was conducted among all forms of TB patients who started treatment from September 2016 to October 2017 in the Jimma Zone, Southwest Ethiopia. The Jimma Zone is located 354 km from Addis Ababa, the capital city of Ethiopia, with a total area of 199,316.18 square kilometres (Jimma Zone health office, 2016). According to the 2017 projected population census, the Jimma Zone had an estimated population of 3,261,371, of which 49.9% were women^14^. In 2016, the Zone had 17 districts and two town administrations. A total of seven public hospitals (five were primary, one general and one specialized), 120 health centers and 494 health posts were registered in the study area during the study period. The hospitals and health centers provided TB diagnostic and treatment services, with health extension workers at health posts actively involved in case holding activities and providing directly observed treatment short-course (DOTS). Non-governmental health facilities such as the Catholic mission and numerous private clinics also provided TB diagnostic and treatment services (the Jimma Zone health office, 2016; the Jimma Town health office, 2016). We used a cross-sectional study design at baseline and record review to identify treatment outcomes.

### TB treatment

In the context of this study, the national guideline for TB, and DR-TB is followed to treat TB^15^. For new TB patients (drug-susceptible TB), the treatment is given for a total of six months. The regimen comprises an intensive phase of daily chemotherapy with rifampicin, isoniazid, pyrazinamide, and ethambutol for two months (2RHZE), followed by a continuation phase treatment with rifampicin and isoniazid for four months (4RH). However, patients with central nervous system and osteo-articular TB require a prolonged continuation phase treatment for 10 months (2RHZE/10RH)^15^.

### Study population and sampling

Eight districts and one town administration were selected from the 17 districts and two town administrations by using a simple random sampling method^16,17^. Subsequently, all TB DOTS sites in the sampled districts and town administration were covered by the study. A total of 1,161 TB patients who were 15 years of age or older and had started anti-TB treatment were consecutively included from the selected study sites. Patients, whose age was less than 15 years, and who could not respond to the interview questions and critically ill patients, were excluded from the study. The minimum sample size was determined by using a single population prevalence formula. A 95% confidence interval, which corresponds to a standard normal deviate of 1.96 (Zα), and a power of 80% (Zβ), which translates to a 0.84 standard normal curve, were considered. A proportion of successful treatment outcome of TB patients (p) from a previous study that showed 88.3%^13^, and a margin error of 4% (D), were taken. Thus, the minimum sample size was calculated to be 506. However, as this study is a sub-study of a larger study, it follows a recently published study that compared community-versus facility-based DOTS in a cohort of 1,161 study participants^18^. We included all the 1,161 study participants in the current study, which was advantageous, as it provides more than an adequate representative population to assess treatment outcome. The sample size was proportionally allocated to the selected public health facilities, based on the previous one-year patient flow. The study participants were consecutively included until the required sample size was achieved.

### Data collection and analysis

A structured questionnaire was developed based on the national and WHO guidelines and previous studies^7,19–23^ (S1 text). The questionnaire was translated to the local language (Afan Oromo) by a professional translator, whose mother tongue is the local language. It was checked for any inconsistencies between the translated version and the original English version of the questions. The translated version of the questionnaire was retranslated to English by another professional, who fluently speaks and writes the local language. It was then pre-tested to check for the clarity and applicability of the questionnaire in the context of the study area. Based on the results of the pre-test, adjustments were made, such as clarifying statements. We recruited experienced data collectors and supervisors, with adequate training provided on data collection and supervision techniques. The principal investigator supervised the entire data collection process. In the first phase of data collection (within the first 15 days of treatment), study participants were consecutively enrolled and interviewed (September 2016 to October 2017). They were followed from the time of enrolment until their treatment outcomes were recorded. In the second phase of data collection, clinical data, including treatment outcomes, were collected from laboratory and TB registers at participating health facilities (April 1 to June 30, 2018).

The collected data were checked for completeness and consistency, and then coded and entered into the EpiData entry client software, version 4.4.3.1. Next, the data were exported to the statistical package for social sciences software (SPSS) version 21 for analysis. Descriptive statistics were computed for the variables.

A total of six questions were asked to assess participants’ knowledge of TB. A correct answer was given a score of one and an incorrect answer was given a score of zero. Subsequently, the total knowledge score and median score were computed. Those with a total score of less than the median value was categorized as having poor knowledge, while those with a total score of greater than or equal to the median value, were classified as having good knowledge. Likewise, a total of 12 questions were asked to assess perceived stigma. A score of one was given for “yes” and a score of “zero” was given for no responses for the questions addressing stigma. Total perceived stigma and median scores were determined. Those with a total score of less than the median value was classified as perceived to not be stigmatized, whereas those with a total score of greater than or equal to the median value was categorized as perceived to be stigmatized.

The different TB treatment outcomes were grouped into: (1) Favorable (treatment completed, cured), and (2) an unfavorable treatment outcome (died, treatment failure, lost to follow-up). Since the final outcome for transferred out and unrecorded cases was unknown, we did not include these as a favorable or unfavorable outcome. To help analyze the association of independent variables with the dependent variable, we used a binary logistic regression analysis. First, a bivariate analysis was performed for each independent variable against the outcome variable (treatment outcomes), and a crude odds ratio (COR) was calculated. A multivariable logistic regression analysis was conducted, including candidate variables in the bi-variable analysis (a p-value of < 0.25) with the outcome variable, and the respective AOR and 95% CI were computed. A p-value < 0.05 was considered statistically significant.

### Operational definitions

The definitions of treatment outcomes used in this study are based on the national and WHO tuberculosis treatment guidelines^21,24,25^ The operational definitions are described below.

**Table:** 

Terms	Operational definitions
Cured	A TB patient with bacteriologically confirmed pulmonary TB at the start of treatment, who was smear- or culture-negative at the end of the treatment, and at least one previous time.
Treatment completed	A TB patient who completed treatment without evidence of smear-or culture- negative at the end of treatment and at least one previous time were negative, either because tests were not done or because results are unavailable.
Treatment failure	A TB patient whose sputum smear or culture is positive at the fifth month or later during the course of treatment.
Lost to follow-up	A TB patient who has been on treatment for at least four weeks, and who discontinued the treatment for eight or more consecutive weeks.
Died	A TB patient who dies from any cause during the course of TB treatment.
Not evaluated	A TB patient for whom treatment outcome was not assigned, which include transferred out and not recorded or unknown treatment outcome.
Favorable treatment outcome	Include TB patients that are “cured” and “treatment-completed”.
Unfavorable treatment outcome	Include “deaths”, “treatment failures” and “lost to follow-up” outcomes.
Total delay	The delay from the onset of TB symptoms to the first start of anti-TB treatment (the sum of patient delay and health system delay).
History of chronic illness	Previous or current chronic disease such as hypertension, diabetes, heart disease, kidney disease, etc., measured by yes or no questions.
HIV status	Whether the study participants were HIV positive or negative, or status unknown. These data were obtained from local registries.
Alcohol use	Whether or not study participants consumed alcohol, assessed with yes or no questions.
Khat chewing	Khat is a flowering, evergreen plant, primarily used in East African countries like Ethiopia, Djibouti, Kenya, Somalia, and Yemen. It is used for its stimulant effects, containing cathine and cathinone. Study participants were asked a yes or no question regarding Khat use.
Medical provider	This term include hospitals, health centers, health posts, private clinics and drug-retail-outlets.
*Woreda*	Woreda is the third level of the administrative division after “zone” and “region” in Ethiopia. It has a defined geographic area, and the population size may range between 50,000 to 200,000.
*Zone*	Zone is defined as the second level of the administrative division in Ethiopia, below “region” and above *woredas*. It has a defined geographic area, and the population size may range between 500,000 to 3 million.
Urban	Refers to the capital of a zone, a woredas well as a town.
Rural	Refers to any areas outside of the “Urban” areas.
Not applicable	Examination of sputum was not required either because the patient was not a pulmonary-TB positive, or the outcome was known at this stage (died, transferred-out).

## Results

### Characteristics of the study participants

The present study assessed treatment outcomes and the associated factors of unfavorable treatment outcomes among TB patients in the Jimma Zone, Southwest Ethiopia. A total of 1,161 patients with all forms of TB participated in the study. Of these, 51.2% were male, 65.9% were married and 39.5% were illiterate. Nearly half (46.9%) of the patients were 25–44 years old. Of the total number of study participants, 63.7% were farmers by occupation, with 50.3% of the participants having a monthly household income of ≤ 1000 Ethiopian Birr, which is equivalent to 29 USD at the current market rate (Table 1).

**Table 1:** Socio-demographic characteristics of the study participants in the Jimma Zone, 2017 (*N* = 1161).

Variables		Frequency	Percentage (%)
Age in years	15–24	365	31.4
	25–44	544	46.9
	45–64	202	17.4
	≥65	50	4.3
Sex	Male	594	51.2
	Female	567	48.8
Marital status	Single	330	28.4
	Married	765	66.1
	Divorced	30	2.6
	Widowed	34	2.9
Educational status	Illiterate	459	39.5
	Read and write only	98	8.4
	Primary school	396	34.1
	Secondary school	132	11.4
	College/University	76	6.5
Occupation	Farmer	739	63.7
	Merchant	74	6.4
	Government or NGO employee	58	5.0
	Daily laborer	88	7.6
	Housewife	19	1.6
	Student	142	12.2
	Unemployed	41	3.5
Religion	Orthodox Christian	160	13.8
	Muslim	939	80.8
	Protestant	61	5.3
	Catholic	1	0.1
Source of household income	Farming	769	66.2
	Monthly salary	59	5.1
	Private/trading	78	6.7
	Daily payment	82	7.1
	Family/relative	152	13.1
	Prefer not to answer	21	1.8
Monthly household income in ETB	≤ 1,000	584	50.3
	1,001–2,500	388	33.4
	2,501–3,500	41	3.5
	> 3,500	90	7.8
	Do not have a regular income	58	5.0

### Study districts and registered treatment outcome categories

Of the total 1,161 patients included in the study, 1,008 (86.9%) had a favorable treatment outcome, 66 (5.7%) had an unfavorable treatment outcome, 34 (2.9%) were transferred out, and 53(4.5%) were not recorded. A majority (18.9%) of the study participants were from the *Goma* district.

The *Omo Nada* district achieved a performance of 90.2%, while the *Sokoru* district scored 89.8%, outperforming the *Limu Kosa* district, which achieved 81.9%. The proportion of death was higher at the *Mana* district (6.2%) compared to *Jimma* Town (1.3%). At *Limu Kosa*, there were a lot of unrecorded cases (11.1%). However, the *Seka Chokorsa* District did not have any unrecorded instances (Table 2).

**Table 2:** Districts and number (percentage) of patients registered in the various treatment outcome categories at the Jimma Zone, Ethiopia, 2017.

District / town administration	Favorable treatment outcome			Unfavorable treatment outcome				Transferred out	Not recorded
	Cured	Treatment completed	Total	Died	Treatment failure	Lost to follow up	Total		
*Goma* (*n* = 220)	67(30.5)	121(55.0)	188(85.5)	10(4.5)	0(0.0)	5(2.3)	15(6.8)	4(1.8)	13(5.9)
*Jimma* (*n* = 157)	48(30.5)	91(58.0)	139(88.5)	2(1.3)	1(0.6)	2(1.3)	5(3.2)	11(7.0)	2(1.3)
*Kersa* (*n* = 122)	36(29.5)	69(56.6)	105(86.1)	5(4.1)	0(0.0)	1(0.8)	6(4.9)	3(2.5)	8(6.5)
*Limu kosa* (*n* = 144)	51(35.4)	67(46.5)	118(81.9)	5(3.5)	2(1.4)	0(0.0)	7(4.9)	3(2.1)	16(11.1)
*Mana* (*n* = 97)	41(42.3)	42(43.3)	83(85.6)	6(6.2)	1(1.0)	1(1.0)	8(8.2)	3(3.1)	3(3.1)
*Omo nada* (*n* = 102)	57(55.9)	35(34.3)	92(90.2)	2(2.0)	1(1.0)	2(2.0)	5(5.0)	2(2.0)	3(2.8)
*Seka chokorsa* (*n* = 120)	48(40.0)	58(48.3)	106(88.3)	7(5.8)	3(2.5)	1(0.9)	11(9.2)	3(2.5)	0(0.0)
*Sokoru* (*n* = 108)	43(39.8)	54(50.0)	97(89.8)	2(1.9)	1(0.9)	2(1.9)	5(4.7)	2(1.9)	4(3.6)
*Tiro afeta* (*n* = 91)	44(48.4)	36(39.6)	80(88.0)	2(2.2)	1(1.1)	1(1.1)	4(4.4)	3(3.3)	4(4.3)
Total (*N* = 1,161)	435(37.5)	573(49.4)	1008(86.9)	41(3.5)	10(0.9)	15(1.3)	66(5.7)	34(2.9)	53(4.5)

### Determinants of unfavorable treatment outcome

During a bivariate analysis, sex, age groups, educational level, alcohol use, Khat chewing, history of chronic illness, HIV status, sputum result at the 2nd or 3rd month, sputum result at the end of the 5th month, and perceived to be stigmatized, were found to be associated with unfavorable treatment outcome. The median time from the onset of symptoms to the start of treatment (total delay) was 35 days (see Table 3).

**Table 3:** Association of socio-demographic and clinical factors with unfavorable treatment outcome among the study participants at the Jimma Zone, Southwest Ethiopia, 2017.

Variables		Treatment outcomes, N*o* (%)		COR (95% CI)	P-values
		Favorable	Unfavorable		
Total delay	Not delayed	505 (50.1)	32 (48.5)	1	
	Delayed	503 (49.9)	34 (51.5)	0.94 [0.57,1.54]	0.799
Sex	Male	507 (50.3)	42 (63.6)	1	
	Female	501 (49.7)	24 (36.4)	1.73 [1.03,2.89]	0.038
Age groups in years	15–24	319 (31.6)	10 (15.2)	1	
	25–44	479 (47.5)	31 (47.0)	0.14 [0.05,0.38]	< 0.001
	45–64	174 (17.3)	17 (25.8)	0.29 [0.13,0.68]	0.004
	≥ 65	36 (3.6)	8 (12.1)	0.44 [0.18,1.10]	0.078
Educational level	Illiterate	396 (39.3)	28 (42.4)	4.67 [0.62,34.88]	0.133
	Read and write only	81 (8.0)	11 (16.7)	8.96 [1.13,71.23]	0.038
	Primary school	344 (34.1)	24 (36.4)	4.61 [0.61,34.63]	0.138
	Secondary	121 (12.0)	2 (3.0)	1.09 [0.10,12.26]	0.944
	College/Uni.	66 (6.5)	1 (1.5)	1	
Monthly household income in ETB	≤ 1,000	504 (50.0)	39 (59.1)	0.91 [0.31,2.66]	0.862
	1,001–2,500	338 (33.5)	21 (31.8)	0.73 [0.24,2.22]	0.579
	2,501-3,500	37 (3.7)	1 (1.5)	0.32 [0.03,2.96]	0.314
	> 3,500	82 (8.1)	1 (1.5)	0.14 [0.02,1.32]	0.086
	No regular income	47 (4.7)	4 (6.1)	1	
Time to reach nearest medical provider min.	≤ 30	690 (68.5)	40 (60.6)	1	
	31–60	241 (23.9)	20 (30.3)	0.74 [0.31,1.81]	0.515
	> 60	77 (7.6)	6 (9.1)	1.07 [0.41,2.75]	0.896
Alcohol use	Yes	75 (7.4)	13 (19.7)	3.05 [1.59,5.85]	0.001
	No	933 (92.6)	53 (80.3)	1	
Khat chewing	Yes	505 (50.1)	44 (66.7)	1.99 [1.18,3.37]	0.010
	No	503 (49.9)	22 (33.3)	1	
History of chronic illness	Yes	36 (3.6)	9 (13.6)	4.26 [1.96,9.28]	< 0.001
	No	972 (96.4)	57 (86.4)	1	
HIV status	Positive	27 (2.7)	8 (12.1)	3.41[1.08,10.74]	0.036
	Negative	912 (90.5)	52 (78.8)	0.66 [0.27,1.58]	0.347
	Unknown	69 (6.8)	6 (9.1)	1	
TB classification	Bacteriologically confirmed	490 (48.6)	33 (50.0)	1	
	Clinically diagnosed	213 (21.1)	19 (28.8)	1.47 [0.77,2.79]	0.241
	Extra PTB	305 (30.3)	14 (21.2)	1.94 [0.95,3.96]	0.067
Sputum result at the 2nd or 3rd month	Negative	452 (44.8)	18 (27.3)	1	
	Positive	12 (1.2)	4 (6.1)	0.61 [0.34,1.09]	0.093
	Not done	27 (2.7)	10 (15.2)	5.07 [1.55,16.55]	0.007
	Not applicable	517 (51.3)	34 (51.5)	5.63 [2.52,12.58]	< 0.001
Sputum result at the end of 5th month	Negative	417 (41.4)	4 (6.1)	0.13 [0.05,0.36]	< 0.001
	Positive	2 (0.2)	3 (4.5)	19.92 [3.23,122.7]	0.001
	Not done	71 (7.0)	20 (30.3)	3.74 [2.07,6.77]	< 0.001
	Not applicable	518 (51.4)	39 (59.1)	1	
Knowledge about TB	Poor	410 (40.7)	26 (39.4)	0.95 [0.57,1.58]	0.837
	Good	598 (59.3)	40 (60.6)	1	
Perceived to be stigmatized	No	328 (32.5)	9 (13.6)	0.33[0.16,0.67]	0.002
	Yes	680 (67.5)	57 (86.4)	1	

Sex, monthly household income, alcohol use, sputum result at the end of the 5th month, and perceived to be stigmatized, remained significant predictors of unfavorable treatment outcome after a multivariable analysis. Female patients were about two times more likely to have an unfavorable treatment outcome compared to male patients (AOR = 1.96, 95% CI 1.06, 3.64). Those patients who used alcohol were about three times more likely to have unfavorable treatment outcome than their counterpart (AOR = 3.16, 95% CI 1.39, 7.15). Patients who had a monthly household income of > 3500 ETB were 96% less likely to have an unfavorable outcome than patients who had no regular income (AOR = 0.04, 95% CI 0.01, 0.45). Patients who perceived themselves not to be stigmatized were 68% less likely to have an unfavorable treatment outcome than their counterparts (AOR = 0.32, 95% CI 0.15, 0.73). (see Table 4).

**Table 4:** Determinants of unfavorable treatment outcome among the study participants at the Jimma Zone, Southwest Ethiopia, 2017.

Variables		Treatment outcomes, N*o* (%)		AOR (95% CI)	P-value
		Favorable	Unfavorable		
Sex	Male	507 (50.3)	42 (63.6)	1	
	Female	501 (49.7)	24 (36.4)	1.96 [1.06,3.64]	0.033
Monthly household income in ETB	≤ 1,000	504 (50.0)	39 (59.1)	0.36 [0.10,1.30]	0.119
	1,001–2,500	338 (33.5)	21 (31.8)	0.30 [0.08,1.14	0.078
	2,501- 3,500	37 (3.7)	1 (1.5)	0.34 [0.03,3.67]	0.376
	> 3,500	82 (8.1)	1 (1.5)	0.04 [0.01,0.45]	0.009
	No regular income	47 (4.7)	4 (6.1)	1	
Alcohol use	Yes	75 (7.4)	13 (19.7)	3.16 [1.39,7.15]	0.006
	No	933 (92.6)	53 (80.3)	1	
Sputum result at the end of the 5th month	Negative	417 (41.4)	4 (6.1)	0.01 [0.00,0.02]	< 0.001
	Positive	2 (0.2)	3 (4.5)	0.11 [0.01,3.67]	0.214
	Not done	71 (7.0)	20 (30.3)	0.04 [0.01,0.42	0.007
	Not applicable	518 (51.4)	39 (59.1)	1	
Perceived to be stigmatized	No	328 (32.5)	9 (13.6)	0.32[0.15,0.73]	0.006
	Yes	680 (67.5)	57 (86.4)	1	

AOR = adjusted odds ratio; COR = crude odds ratio; CI = confidence interval; 1 = Reference group.

## Discussion

In this study, we assessed treatment outcome and the determinants of unfavorable treatment outcome among TB patients in the Jimma Zone, Southwest Ethiopia. The study has shown that being a female patient, having a poor household income and being an alcohol consumer may decrease the chance of having TB treatment success. For the entire zone, only 86.9% of the patients had a successful treatment outcome. Our results fell short of both the WHO’s and the national goal of successfully treating more than 90% of cases of TB discovered^3,26^. This difference might be due to a substantial variation of unfavorable treatment outcome observed across districts, zones and regions in Ethiopia^27^, which suggests that the Zonal TB control program has to take a variety of measures to improve the treatment success rate.

An unfavorable treatment outcome was significantly associated with socio-demographic factors, such as sex, economic factors, such as household income, behavioral factors, such as alcohol use, as well social factors, such as stigma in the community. Our study revealed that women were more likely to have unfavorable treatment outcome compared to men. This finding is similar to former studies conducted in Ethiopia and other African countries like Nigeria^28–30^. Our finding could be explained by the fact that economic status may influence treatment outcome, as women may have less access to financial resources in the household. Cultural beliefs about the early symptoms of TB (e.g., interpreted as another illness)^31^, and perceived stigma, are barriers of early care seeking and treatment adherence for a majority of female patients^32^. As reported from several countries^32–35^, economic dependence and constraints associated with religious or cultural norms can contribute to social inequality among women. This inequality may result in a lack of power to decide whether to use money (e.g., for transport, diagnostic medical procedures), and how to prioritize the use of time (e.g., DOTS being a time-consuming daily treatment). Several studies from a variety of low-and middle income countries show that these are factors that may reduce accessibility to medical services, and affect treatment adherence^32–35^. This finding underscores the importance of recognizing gender differences in an individual’s TB treatment outcome. To help increase treatment adherence and enhance the outcomes of TB patients, it appears essential to address the specific needs of women through socioeconomic support interventions, or by modifying current barriers (excessive use of time on a daily basis, indirect/direct costs) inherent in the TB control program^36^. Furthermore, research shows that the use of adherence interventions, including patient education and counselling, reminders and digital health technologies, significantly improve TB treatment outcomes^37^. Developing well-crafted Information, Education and Communication (IEC) strategies, and Behavioral Change Communication (BCC) interventions that target women, could be helpful for treatment adherence. Several media platforms, including local radio and text messages, can be used to address information gaps regarding both early symptoms and treatment adherence^38,39^.

However, the available evidence regarding the influence of sex in treatment outcomes has revealed inconsistent findings. Our findings differ from many previous studies conducted in Ethiopia and elsewhere, which found that male patients were more likely than female patients to have unfavorable treatment outcomes^9,11,30,40,41^. One reason for this could be that men are more exposed to TB infections associated with behavioral factors, such as alcohol use or being a smoker (which affects treatment adherence), compared to women^42,43^. In addition, men and women have different societal roles and occupations (such as men often being a daily laborer), which can affect not only their risk of exposure to TB, but also their access to medical care^44,45^. The observed difference in our findings might also be related to differences in the study settings, study methods, and socio-economic status of the population in the respective studies in Ethiopia and elsewhere. It is also an established fact that the gender-based social effect on decision-making related to health might differ across cultures and settings^46^.

Perceived stigma was identified as an independent predictor of unfavorable treatment outcome in our study. TB stigma is a major barrier to early diagnosis and successful treatment completion. A systematic review in low-, middle- and high-income countries showed that perceived TB stigma resulted in delays in diagnosis and poor treatment adherence^47^. Previous studies from Ethiopia and elsewhere has shown that TB/HIV/AIDS-related stigma, often stemming from high co-infection rates where people associate TB as the initial symptom of HIV/AIDS or consider them as the same disease, alongside the fear of TB transmission due to misconceptions about its spread, were identified as the main sources of TB stigma^48–52^. Such misconceptions may lead patients to be afraid of disclosing themselves as TB patients^31^. TB patients on treatment may avoid seeking treatment or default from treatment because of a fear of discrimination by others, and a lack of support from their families and/or community^31^. The fear of unemployment and isolation has also been found to affect individuals’ disclosure about TB^48,52^. Thus, TB stigma tends to result in poor treatment adherence, and subsequently to poor prognosis^47,48^. Earlier studies from Ethiopia showed that perceived stigma was even higher among TB patients than their families and general population^48,52^. Because of the strong stigma attached to TB, patients could be denied housing and a lack of support from their families and community^48,52^. The Oromia region (where our study area is located) exhibited the highest score of TB stigma when compared to other regions of Ethiopia^48^. Studies show that, for example, patients living in urban areas like Addis Ababa (capital city) have a higher knowledge about TB, and a lower stigma score^48,52^. Hence, it is important to assess the illness perception of TB patients to identify their vulnerability to unfavorable treatment outcome. Many health-care providers disregard patients’ thoughts and opinions about their illness, and do not recognize the reasons for different illness perceptions, misconceptions or rationalities that may lead to poor adherence to TB treatment^50,53^. The early recognition of a patient’s illness perception will provide knowledge that is important to planning and implementing patient-centered care related to TB-related stigma, and an adherence to TB treatment in general^50,53^. Previous research indicates that establishing patient and peer support groups, implementing TB education or support initiatives aimed at healthcare providers, TB patients, and individuals at risk, could potentially mitigate TB stigma^47,54^. An engagement of different stakeholders (multidisciplinary approach), such as psychologists’ and/or sociologists’, is suggested to reduce stigma due to TB^47,54^. As TB-related stigma contributes to a low effectiveness of the existing TB control program, understanding and addressing the local perceptions of TB is essential to increase the treatment success rate^50,52,55,56^.

In this study, a poor household income was associated with an unfavorable treatment outcome. In line with our findings, several previous studies document the impact of a poor socio-economic status on TB treatment outcomes in both low- and high-income countries                  ^27,57–59^. A low household income may affect the patients’ adherence to daily treatment with DOTS because of expenses related to, for example, transport and the use of time (e.g., daily laborers not being able to meet up in the morning at regular meeting places to find a job for the day)^60,61^. In addition to long treatment regimens, excessive psycho-social stress may be one possible biological reason behind an increased chance of poor treatment outcome among patients with a low household income. Studies indicate that a poor treatment outcome may result from excessive stress, as financial insecurity may negatively affect immune functions through long-term stress effects on the “Hypothalamic-Pituitary-Adrenal axis”^62,63^. In addition, patients with a poor household income may not get adequate nutrition, which may result in a poor immune function^64^. As evidenced in earlier studies, proper nutrition is associated with increased immune system strength^65,66^ and improved clinical outcomes among TB patients^67^.

Alcohol use was another predictor of TB treatment outcome in our study. Patients who used alcohol were more likely to have an unfavorable treatment outcome than patients who did not use alcohol. This finding is in line with previous studies’ results. A systematic review and meta-analysis revealed that non-alcohol drinkers were two times more likely to have a favourable treatment outcome than their counterparts^68^. In another systematic review, alcohol use was identified as a predictor of treatment outcomes for both drug-susceptible (DS) and multi-drug resistance (MDR)-TB, whereby alcohol use was associated with a higher risk of unfavourable treatment outcome^69^. A former study in Ethiopia reported that alcohol intake was a significant risk factor for non-adherence to TB treatment, and related to poor treatment outcome^70^. Similarly, a study from Kenya found that alcohol use was significantly associated with the interruption of TB treatment^71^. This may be because patients who use alcohol on a regular basis are more likely to interrupt their TB treatment regimen, as well as more likely to forget following their treatment when they are intoxicated.

Additionally, the adverse interactions between alcohol and anti-TB medications may lead patients to discontinue their TB treatment regimen due to unwanted effects^71^. This highlights the necessity for tailored psycho-social support interventions to enhance treatment adherence and promote better treatment outcomes among TB patients who consume alcohol^38^.

This study has strengths and limitations. The strength of the study is that we included a relatively large sample size compared to sample sizes used in other similar studies in Ethiopia. Using a larger sample size provides a more than adequate representative population to address the research objectives. Regarding potential limitations, the study was only carried out in government health facilities; hence, the findings cannot be generalized to all TB patients in the study area. There are several private health care facilities where patients get anti-TB treatment, although the treatment outcome of patients attending private health-care facilities may be different. However, since these facilities are fewer in number, there is minimal chance of selection bias, thus the current findings are unlikely to be affected. Our study was conducted prior to the COVID-19 pandemic, and it is possible that treatment outcomes may differ during this period. Therefore, future study is required to assess the effect of COVID-19 pandemic on the TB treatment outcome in the study area. Although we used a pretested tool, it has not been validated, which requires careful interpretation of the findings of variables such as TB knowledge assessment and perceived stigma.

## Conclusion

The treatment success rate observed in this study falls below the WHO’s target of achieving a treatment success rate of > 90% for detected TB cases. This suggests that there were patients being non-adherent to the prescribed anti-TB drugs. Being female, alcohol consumption, low household income, and perceived stigma were all linked to unfavorable treatment outcomes. These results underscore the necessity of developing tailored strategies to enhance treatment outcomes among these specific patient groups.

Various interventions could enhance treatment outcomes. It is crucial to address the unique needs of women and patients with low income through socioeconomic support initiatives or by addressing existing barriers, such as excessive time demands inherent in TB control programs. Patients who consume alcohol may require extra support and follow-up. Furthermore, exploring and addressing local illness perceptions; perceptions that may cause stigma and potentially delay diagnosis, or that results in poor treatment adherence seems important both on the individual and society level. Overall, it appears important to provide individually tailored treatment-adherence counseling and follow-up, as each patient may face unique, but often interrelated barriers.

## Electronic supplementary material

Below is the link to the electronic supplementary material.

Supplementary Material 1

## Data Availability

All data sets generated or analyzed during the current study are available upon request from the corresponding author.

## Abbreviations

AOR: Adjusted odds ratio
COR: Crude odds ratio
DOTS: Directly observed treatment short-course
DS: Drug-susceptible
MDR: Multi-drug resistance
RH: Rifampicin and isoniazid
RHZE: Rifampicin, isoniazid, pyrazinamide and ethambutol
SPSS: Statistical package for social sciences software
TB: Tuberculosis
WHO: World Health Organization
